# The complete mitochondrial genome of *Aucklandia lappa* Decne. (Asteraceae, *Aucklandia* Falc.)

**DOI:** 10.1080/23802359.2021.1914524

**Published:** 2021-05-19

**Authors:** Yue-Yue Song, Ting-Ting Zhang, Hui Tang, Liang Xu, Yan-Ping Xing, Rong Zhao, Gui-Hua Bao, Sheng-Nan Li, Da-Chuan Zhang, Yang Qiao, Yan-Yun Yang, Wu-Liji Ao, Ting-Guo Kang

**Affiliations:** a School of Pharmacy, Liaoning University of Traditional Chinese Medicine, Dalian, China; b School of Mongol Medicine, Inner Mongolia University for Nationalities, Tongliao, China

**Keywords:** Complete mitochondrial genome, phylogenetic tree, *Aucklandia lappa*

## Abstract

The complete mitochondrial genome of *Aucklandia lappa* was sequenced for the first time. The mitochondrial genome length was 320,439 bp, with 45.05% GC contents. There were 67 genes annotated, including 31 known protein-coding genes, 25 tRNAs, and six rRNAs. The maximum likelihood method was used to establish the phylogenetic tree of 37 species. Results have shown that *A. lappa* and *Arctium lappa* were sister groups. It reveals the genetic relationship between different species and provides a theoretical basis for the establishment of a classification system.

*Aucklandia lappa* Decne. is a perennial herb belonging to *Aucklandia* Falc., synonymous with *Saussurea costus*, *Saussurea lappa*, and *Aucklandia costus* (Rohit et al. 2020). Native to Yunnan Province in China, *A. lappa*, also known as ‘Guangmuxiang,’ later is mainly imported from places like Egypt, India and Nepal. In 1935, the herb was first introduced from India and successfully grown in Yunnan. Widely acclaimed for its superior quality, the species was given a new name ‘Yunmuxiang.’ The plant is widely cultivated in Sichuan (Mount Emei), Yunnan (Weixi, Kunming), Guangxi and Guizhou (Guiyang, Dushan). The root of *A. lappa* has medicinal value, mainly used to treat stomach diseases. The paste is applied to the inflamed area to relieve pain. The chemical constituents are terpenoids, steroids, phenylpropanoids, lignans, flavonoids and so on (Benedetto et al. 2019; Rohit et al. 2020). According to the Chinese pharmacopeia (National Pharmacopeia Commission 2020), Radix Aucklandiae for medicinal use should contain at least 15% total costunolide (C_15_H_20_O_2_) and dehydrocostus lactone (C_5_H_18_O_2_). Studies have shown that costunolide and dehydrocostus lactone are potential anticancer drugs (Chen et al. 1995; Jeong et al. 2002; Cho et al. 2004). So far, the whole chloroplast genome of *A. lappa* has been sequenced (Xu et al. 2019).

Fresh leaves of *A. lappa* were collected from Weining County, Guizhou Province, China (E 104°32′46″, N 26°47′15″). The voucher specimen was identified by Professor Tingguo Kang (Liaoning University of Chinese Medicine, Dalian, China). The voucher specimen and genomic DNA were deposited at the herbarium of Liaoning University of Chinese Medicine (Liang Xu 861364054@qq.com, *A. lappa* number: 10162201125069LY). Total mitochondrial genomic DNA was extracted from fresh leaves and sequenced by Illumina NovaSeq 6000 and PacBio Sequel II platform. Assemble the sequencing data with GetOrganelle v1.6.4 (Jin et al. 2020) and correct the bases with Pilon v1.22 to get the final genome sequence of mitochondria. The protein coding sequences of mitochondria were compared with known protein databases (NR, Swiss-Prot, eggNOG, KEGG, GO) to predict protein-coding genes.

The mitochondrial genome of *A. lappa* was 320,439 bp in length with the typical circular structure, and GC content was 45.05%. There were 67 genes annotated, including 31 known protein-coding genes, 25 tRNAs, and six rRNAs. The accumulated length of the coding gene was 29,127 bp, and the length of the coding region accounted for 9.09% of the genome. In addition, we found that eight genes (*nad5, nad2, ccmFc, cox2, nad1, nad4, nad7, rps3*) contained 20 introns.

As we all know, phylogenetic tree is used to describe the evolutionary relationship between species. We selected the complete mitochondrial genome of 37 species (including *A. lappa*) to construct the phylogenetic tree, using the maximum-likelihood method with the model GTR + I + G (Figure 1). Among them, *Ginkgo biloba* as the outgroup. The phylogenetic tree showed that *A. lappa* and *Arctium lappa* were sister groups and *Ginkgo biloba* was far from the other species. The whole mitochondrial genome sequencing of *A. lappa* provides the conditions and theoretical basis for its application in plant phylogeny. At present, there are few mitochondrial genomes available in Asteraceae. These data will provide a basis to analyze the phylogenetic position of *A. lappa*.

**Figure 1.**
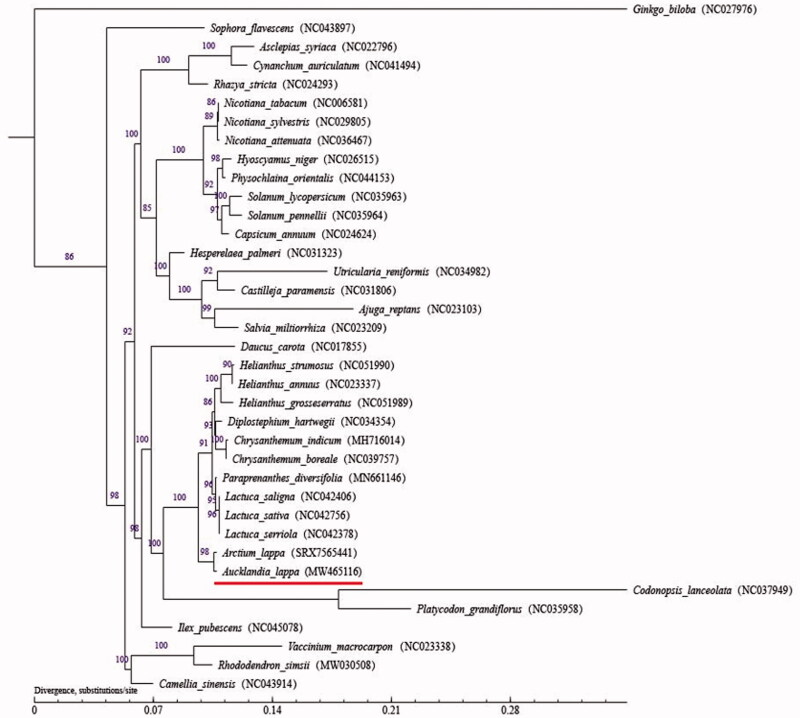
Maximum-likelihood (ML) phylogenetic tree of *Aucklandia lappa* and 36 other species. Numbers above the branches indicate the bootstrap values from ML analyses.

## Data Availability

The genome sequence data that support the findings of this study are openly available in GenBank of NCBI at (https://www.ncbi.nlm.nih.gov/) under the accession NO. MW465116. The associated BioProject, SRA, and Bio-Sample numbers are PRJNA690192, SRX9818099 (PACBIO_SMRT), SRX9818098 (ILLUMINA) and SAMN17245959, respectively.
